# Transcription Factor KLF7 Promotes Osteoclast Differentiation by Suppressing HO-1

**DOI:** 10.3389/fgene.2022.798433

**Published:** 2022-03-28

**Authors:** Changhong Chen, Fei Hu, Shichang Miao, Liping Sun, Yajun Jiao, Mingwei Xu, Xin Huang, Ying Yang, Rongkui Zhou

**Affiliations:** Department of Orthopedics and Injury, Jiangyin Hospital Affiliated to Nanjing University of Chinese Medicine, Jiangyin, China

**Keywords:** osteoporosis, osteoclast genesis, HO-1, KLF7, transcription factor

## Abstract

**Background:** Osteoporosis is a common orthopedic disease with high prevalence in patients older than 50 years. Osteoporosis is often detected only after the fracture and is hard to treat. Therefore, it is of great significance to explore the molecular mechanism of the occurrence of osteoporosis.

**Methods:** The expression of Heme oxygenase-1 (HO-1) in people with different bone mineral density (BMD) was analyzed based on public databases. GenHacncer and JASPAR databases were adopted to search and verify the upstream transcription factor of HO-1. qRT-PCR, western blot and tartrate-resistant acid phosphatase assays were performed to explore the impact of HO-1 and Kruppel-like factor 7 (KLF7) on osteoclast differentiation. Chromatin immunoprecipitation (ChIP) assay confirmed the binding relationship between KLF7 and HO-1. Finally, Hemin, the agonist of HO-1, was applied in rescue assays, thereby verifying the mechanism of KLF7 modulating osteoclast differentiation by HO-1.

**Results:** Bioinformatics analysis revealed that HO-1 was highly-expressed while KLF7 lowly-expressed in people with high BMD. Besides, a potential binding site of KLF7 was found on the promoter region of HO-1. ChIP assay further manifested the targeting relationship between HO-1 and KLF7. Western blot and TRAP staining unveiled that osteoclast differentiation was suppressed by HO-1, while facilitated by KLF7. Rescue experiments indicated that over-expressed HO-1 could reverse of the promoting effect of KLF7 on osteoclast differentiation.

**Conclusion:** The study confirmed that osteoclast differentiation was promoted by KLF7 constraining HO-1, thereby facilitating osteoporosis. The cognation of the pathogenesis of osteoporosis was further enriched. New treatment could be developed on this basis.

## 1 Introduction

Osteoporosis is a common orthopedic disease, which is characterized by low bone mineral density (BMD), the deterioration of bone microarchitecture and the increase of fracture occurrence (Langdahl, 2021). According to the statistics from Strom’s team (Ström et al., 2011), there have been approximately 200 million people suffering from osteoporosis worldwide. Osteoporosis is mainly caused by a disorder of bone renewal and repair. In healthy human bodies, there is a balance that osteoclasts absorb old bone and stimulate osteoblast to synthesize collagen and form new bones, thereby maintaining the bone renewal and the health of bone tissue (Ukon et al., 2019). With the increase of people’s age, such balance is broken, resulting in a decrease in BMD and the fragility of bone tissue, thereby causing fracture (Ukon et al., 2019). Accordingly, it is particularly important to further explore the molecular mechanisms influencing the development of osteoporosis based on the above mechanisms.

Heme oxygenase-1 (HO-1) is known as a negative regulator of osteoclast differentiation and is involved in the process of bone renewal and repair. Generally, Hrf2/HO-1 pathway is considered to be a key pathway that influences the occurrence of osteoporosis (Han et al., 2019). Previous studies indicated that melatonin can activate Nrf2/HO-1 pathway to decrease oxidative damage, so as to inhibit the occurrence of osteoporosis (Ma et al., 2020). Nrf2/HO-1 was also found to improve and relieve the symptoms of osteoporosis by inhibiting inflammatory responses and accelerating the formation of osteoblast (Xu et al., 2019; Liu et al., 2021). Additionally, Ke et al. (2015) reported that the amount and viability of osteoclasts were significantly increased in mice with HO-1 deficiency. The content of reactive oxygen in serum of these mice is significantly higher than that of normal mice. Florczyk-Soluch et al. (2018) also given the negative regulation of HO-1 on osteoclasts. Hence, the exploration on the related factors affecting HO-1 expression is of great value for the research on the pathogenesis of osteoporosis.

Kruppel-like factor 7 (KLF7) is a transcription factor (TF) that accelerates the regeneration in the central nervous system axon (Galvao et al., 2018). It was found to regulate the expression of its target genes in various diseases (Guan et al., 2019; Li et al., 2021). For instance, KLF7 facilitates the growth and metastasis of pancreatic cancer via upregulating the expression of ISG gene (Gupta et al., 2020). Besides, it was revealed that KLF7 is also involved in the occurrence of orthopedic diseases. For example, a study manifested that the overexpression of KLF7 in bone marrow stromal stem cells can promote the regeneration of sciatic nerve (Li et al., 2019). Recently, a bioinformatics study suggested that KLF7 can affect patient’s BMD, which makes it a key target of osteoporosis (Chen et al., 2017). Due to the limitations of bioinformatic studies, the biological role of KLF7 in osteoporosis has not been fully elucidated yet. Taken together, both HO-1 and KLF7 are key genes that impact the growth of bones. Further studies on them therefore will benefit the elucidation of molecular mechanism that causes the occurrence of osteoporosis.

Based on previous studies and bioinformatics results, it could be postulated that HO-1 and KLF7 could regulate the osteoclast differentiation. Molecular and cell experiments were performed to prove that KLF7 accelerates osteoclast differentiation by suppressing HO-1, thereby the development of osteoporosis was affected. The result of this study disclosed the molecular mechanism of KLF7/HO-1 in osteoporosis, which provides theoretical basis for clinical treatment and drug development of osteoporosis.

## 2 Materials and Methods

### 2.1 Cell Lines and Cell Culture

Murine mononuclear macrophage leukemia cell line RAW264.7 (BNCC337875) was purchased from BeNa Culture Collection (BNBIO, China). The cells were cultured in 90% Dulbecco’s modified eagle medium (DMEM) + 10% fetal bovine serum (FBS)and then placed in an incubator with 5% CO_2_ at a constant temperature of 37°C. Recombinant mouse macrophage colony stimulating factor (M-CSF, MedChemExpress, USA) and recombinant mouse receptor activator of nuclear factor kappa-B ligand (RANKL, MedChemExpress, USA) proteins were used to activate RAW264.7 to differentiate into osteoclasts. In short, after the cells in the medium have grown to an appropriate level, the medium was replaced with the medium containing 30 ng/ml M-CSF and 50 ng/ml RANKL. Cells were then used for subsequent assays after being incubated for an appropriate time. Hemin (HO-1 activator) was added into the medium at a final concentration of 10 μM. After 8 h of culture, M-SCF and RANKL were used to activate macrophage differentiation in accordance with the above processes.

### 2.2 Bioinformatics Analysis

Osteoporosis-related gene expression data of blood leukocytes were downloaded from GSE56815 dataset (https://www.ncbi.nlm.nih.gov/gds/), including data of 40 females with low BMD and 40 females with high BMD. The subjects were classified into high and low BMD groups according to their BMDs. Then limma package was used for differential expression analysis and data standardization. Gene expression was visualized using Graphpad Prism. T-test was utilized to analyze the significance of the differences between gene expression in the two groups.

TFs involving in the upstream regulation of HO-1 were predicted using GenHancer database (https://www.genecards.org/Guide/GeneCard#enhancers). These predicted TFs were intersected with differentially expressed TFs in osteoporosis. The expression of acquired TFs and HO-1 were subjected to Pearson correlation analysis. Thereafter, the gene significantly correlated with HO-1 was adopted as the object of the study.

Jaspar database (http://jaspar.genereg.net/) was employed to predict the binding relationship between the predicted target TF and HO-1. Firstly, the 5’ end sequence extending from the transcription start site (TSS) region at 2,000 bp in the upstream of HO-1 gene was downloaded from National Center of Biotechnology Information (NCBI) database (https://www.ncbi.nlm.nih.gov/). Thereafter, this sequence was compared with the TF in Jaspar database. The result was determined as the possible binding site on the TF.

### 2.3 Cell Transfection

si-HO-1, oe-KLF7/si-KLF7 and their negative controls oe-NC/si-NC were procured from Gene Pharma (Gene Pharma, China). 5 ul of Lipofectamine3000 (Themo Fisher, USA) was used to transfect 50 nM si-HO-1 and oe-KLF7/si-KLF7 into cells. Successfully transfected cells were collected after 72 h.

### 2.4 Tartrate-Resistant Acid Phosphatase Staining

The treated cells were collected and inoculated into 24-well plate (1.5×10^4^/well), followed by a fixation with 10% formaldehyde solution. TRAP staining kit (Sigma-Aldrich, USA) was applied to stain the inoculated cells, after which the stained cells were observed under a microscope. A single colony with more than 3 cells whose nucleus was stained (TRAP-positive) was counted as an osteoclast.

### 2.5 RNA Extraction and Quantitative PCR Detection

TRIzol kit (Invitrogen, USA) was used for the extraction of total RNA from the cells based on the instructions of the manufacturer. The concentrations of extracted RNA were assessed utilizing NanoDrop™ One (Thermo Fisher, USA) and then diluted to an equal level. Afterwards, QuantiTect Reverse Transcription Kit (Qiagen, German) was employed to reversely transcribe RNA into cDNA which were then used for qPCR detection proceeding with SYBR™ Green PCR Master Mix (Thermo Fisher, USA). In brief, 1 ng cDNA, appropriate dose of primers, ddH_2_O and SYBR™ Green PCR Master Mix were mixed up. Then the mixture was added into LightCycler® 480 (Roche, UK) for qPCR detection. GAPDH was used as internal reference of the obtained data. The relative expressions were calculated using 2^−ΔΔCT^ method for subsequent analyses. Table 1 presented the sequences of primers used.

**TABLE 1:** Primer sets for qPCR assay.

Primer sets	Sequence (5′-3′)
HO-1	
Forward	AAG​CCG​AGA​ATG​CTG​AGT​TCA
Reverse	GCC​GTG​TAG​ATA​TGG​TAC​AAG​GA
KLF7	
Forward	CTC​ACG​AGG​CAC​TAC​AGG​AAA​C
Reverse	TGG​CAA​CTC​TGG​CCT​TTC​GGT​T
GAPDH	
Forward	GGA​GCG​AGA​TCC​CTC​CAA​AAT
Reverse	GGC​TGT​TGT​CAT​ACT​TCT​CAT​GG

### 2.6 Western Blot Assay

After the cells were treated in accordance with experimental design, they were collected for the extraction of total proteins using radioimmunoprecipitation assay (RIPA) lysis buffer. The content of total protein was measured using bicinchoninic acid (BCA) kit. After the proteins were extracted, 30 μg total proteins were placed on sodium dodecyl sulfate polyacrylamide gel electrophoresis (SDS-PAGE) for isolation. Thereafter, the proteins were transferred onto a polyvinylidene fluoride (PVDF) which was then blocked with 5% nonfat-dried milk. Afterwards, the membrane was sequentially incubated with primary anti-HO-1 (Abcam, USA), anti-GAPDH (Abcam, USA), anti-NFAT2 (CST, USA), anti-TRAP (Abcam, USA), anti-CTSK, anti-KLF7 (Invitrogen, USA) and corresponding secondary antibody (CST, USA). The concentration of the proteins was gauged utilizing electrochemiluminescence (ECL) luminescence reagent. Each assay was repeated three times.

### 2.7 Chromatin Immunoprecipitation

First of all, RAW264.7 cells were transfected using pCMV-Myc-KLF7. After 24 h of transfection, anti-Myc antibody (Invitrogen, USA) and corresponding Simple ChIP enzymatic chromatin IP kit (CST, USA) were subjected to ChIP assay following the instructions of the manufacturer. In the end, depurated DNA was detected using qPCR. The primers used for detections were as follows (Table 2).

**TABLE 2:** Primer sets for ChIP-qPCR assay.

Primer sets	Sequence (5′-3′)
KLF7 upstream	
Forward	GGC​GAG​CTA​TTT​TTA​GAG​GGC​T
Reverse	GCA​TCT​AGT​GGA​GGG​TCG​GAG

### 2.8 Statistics Analysis

All data in this study were denoted as MEAN ± standard deviation (SD). GraphPad Prism and R software were adopted for analysis and drawing. Analysis of variance and Student’s *T*-test was utilized for differential evaluations among groups. *p* < 0.05 denoted a statistical significance.

## 3 Results

### 3.1 HO-1 Suppresses Osteoclast Differentiation

**FIGURE 1:**
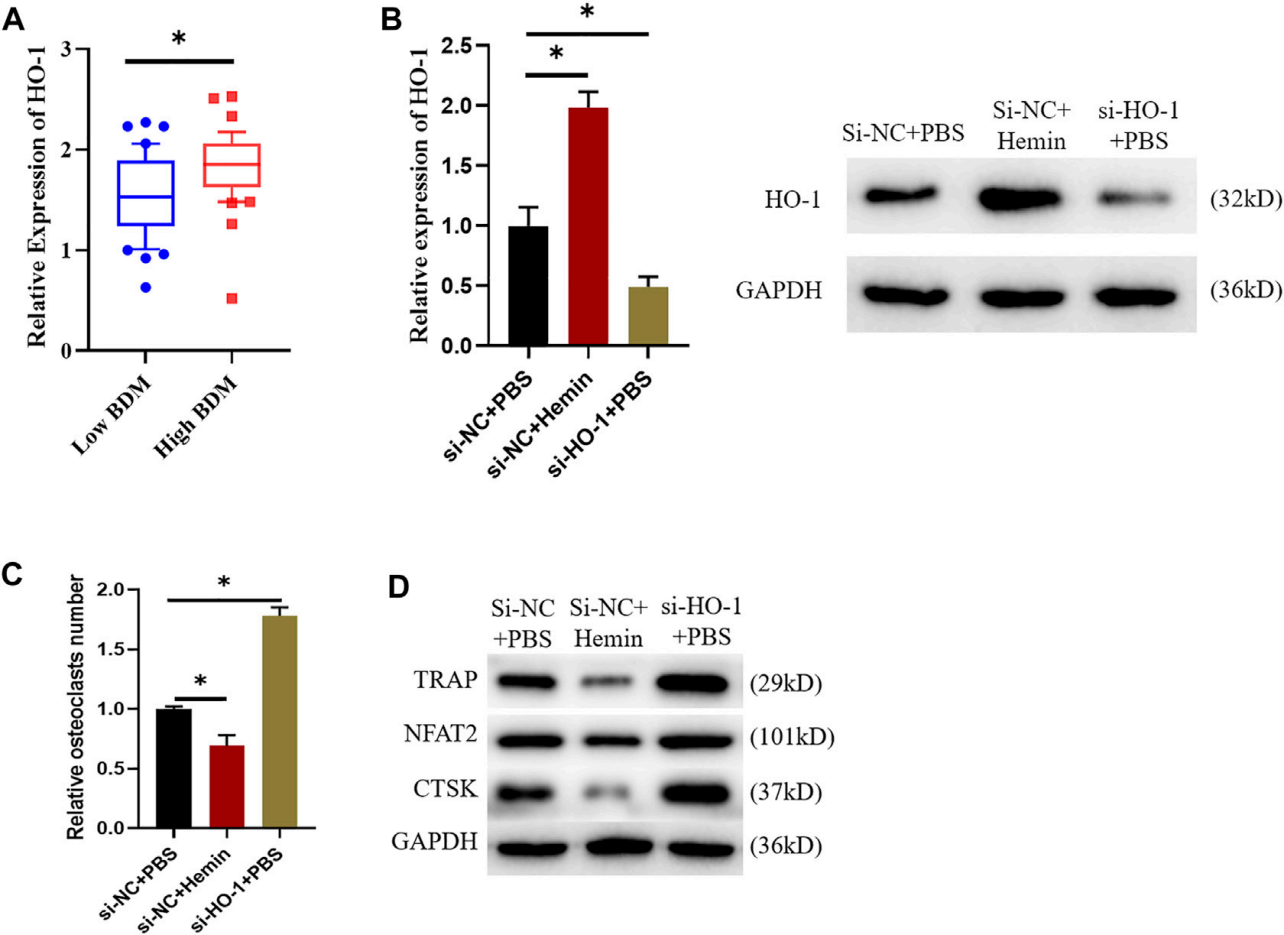
HO-1 can suppress osteoclast differentiation. **(A)** Differences in HO-1 expression level between low and high BDM female. **(B)** Changes of HO-1 mRNA and protein expression levels after si-HO-1 and hemin treatment. **(C)** TRAP staining shows that HO-1 could affect osteoclast differentiation. **(D)** Changes of expression levels of proteins related to osteoclast differentiation after HO-1 silencing and hemin treatment. *p < 0.05.

Firstly, patients’ gene expression data in GSE56815 dataset were downloaded. It was found that the expression of HO-1 was remarkably up-regulated in females with high BMD in comparison with the ones with low BMD. (Figure 1A). In combination with previous studies (Han et al., 2019), we posited that HO-1 expression in blood mononuclear cells somehow affected the BMD of the subjects. Accordingly, raw264.7 was selected for the osteoclast differentiation assay. Group settings were as follows: si-NC + PBS, si-NC + hemin and si-HO-1+PBS groups. Of them, hemin is a kind of heme metabolite, which is considered to promote HO-1 expression (Zwerina et al., 2005). Firstly, we verified the promoting effect of hemin on HO-expression and the transfection efficiency of si-HO-1. It was suggested that HO-1 was highly-expressed in RAW264.7 cells after being treated with hemin while expression of HO-1 was dramatically downregulated after being treated with si-HO-1 (Figure 1B). TRAP staining exhibited that the differentiation ability of osteoclasts after treatment with Hemin was diminished while knockdown on HO-1 expression resulted in an enhancement on such ability (Figure 1C). Finally, we employed western blot to validate the expression of the osteoclasts markers protein. The results revealed that the expression levels of TRAP, NFAT2, and CTSK protein were reduced after Hemin treatment. When HO-1 expression was knock down, the expression levels of TRAP, NFAT2, and CTSK protein were increased (Figure 1D). The above results indicated that HO-1 was highly expressed in high-density people. The promotion of HO-1 expression would inhibit osteoclast differentiation, while knocking down HO-1 promoted osteoclast differentiation.

### 3.2 KLF7 is an Upstream Regulator of HO-1

**FIGURE 2:**
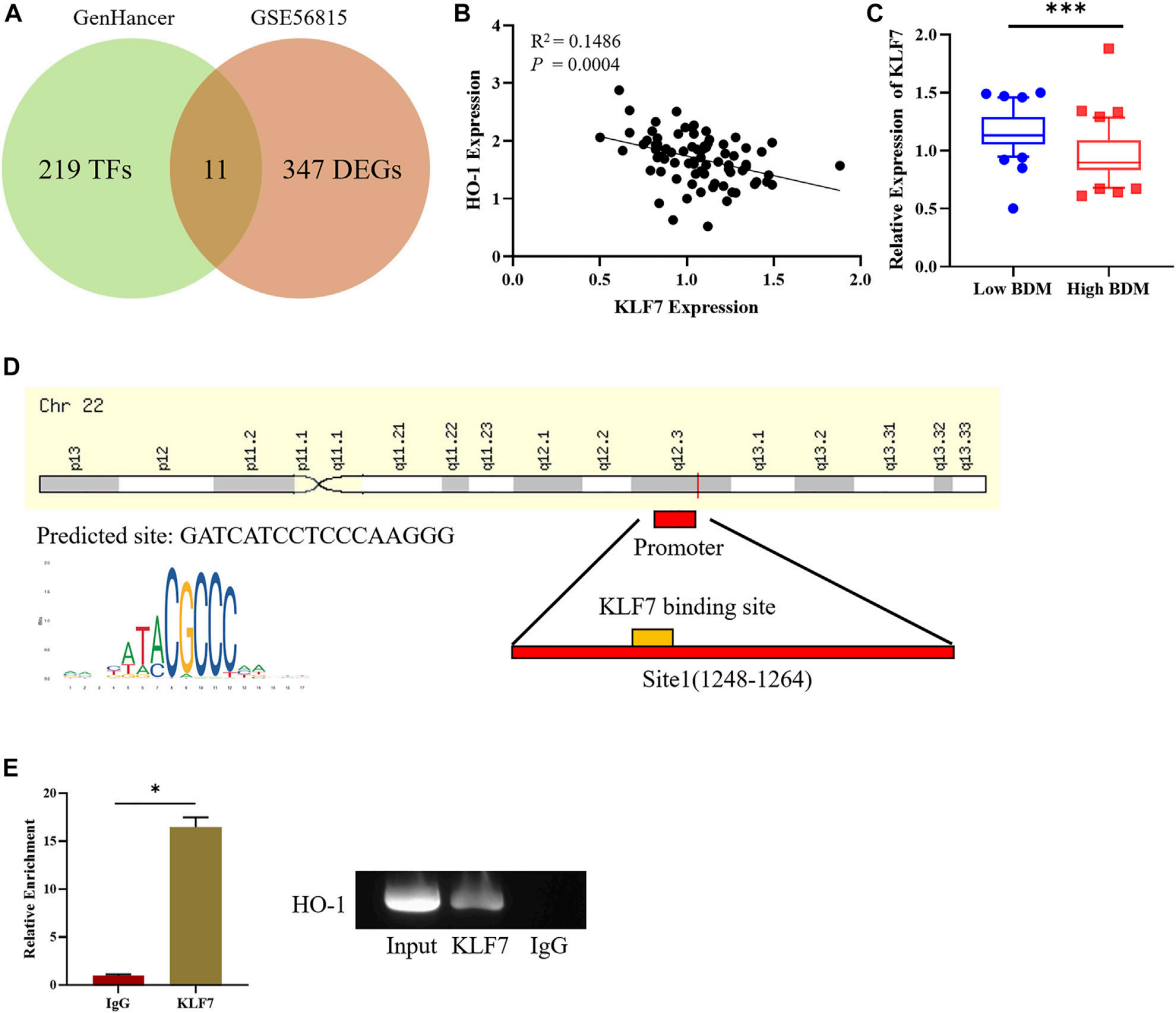
KLF7 is an upstream regulator of HO-1. **(A)** Venn plot for screening DE-TFs in osteoporosis. **(B)** Correlation analysis between KLF7 and HO-1 expressions. **(C)** KLF7 expression in high/low BDM population. **(D)** Predicted KLF7 binding sites on HO-1 TSS. **(E)** ChIP and qPCR assays of the enrichment of KLF7 on the HO-1 promoter region. *p < 0.05, ***p < 0.001.

To dissect the factor that affects HO-1 expression and explore potential therapeutic target of osteoporosis, we used bioinformatics approaches to investigate the regulator in the upstream of HO-1. GenHancer was first adopted to predict the TFs that might affect expression of HO-1. Then the expression data of GSE56815 were downloaded, and 358 differentially expressed genes were screen out (Figure 2A). Differentially expressed genes and possible TFs were intersected. Thereafter a total of 11 differentially expressed TFs were obtained (Figure 2A). Subsequently, correlation analysis was performed on 11 TFs and HO-1. The result suggested a negative correlation between the expression of KLF7 and HO-1 (Figure 2B). Then the expression of KLF7 were tested in people with low and high BMD. The result indicated that KLF7 was significantly lowly-expressed in people with high BMD (Figure 2C). After the TF interacting with KLF7 was found, there was a binding site of KLF7 on the promoter of HO-1 detected by Jaspar database (Figure 2D). The binding relationship of KLF7 to the HO-1 gene promoter was then verified by ChIP assay, and the results implied that KLF7 could bind the HO-1 promoter (Figure 2E). All the above results revealed that KLF7 was an upstream regulator of HO-1. The expression of KLF7 and HO-1 was negatively correlated, where KLF7 negatively regulated the expression of HO-1.

### 3.3 Highly-Expressed KLF7 Can Promote Osteoclast Differentiation

**FIGURE 3:**
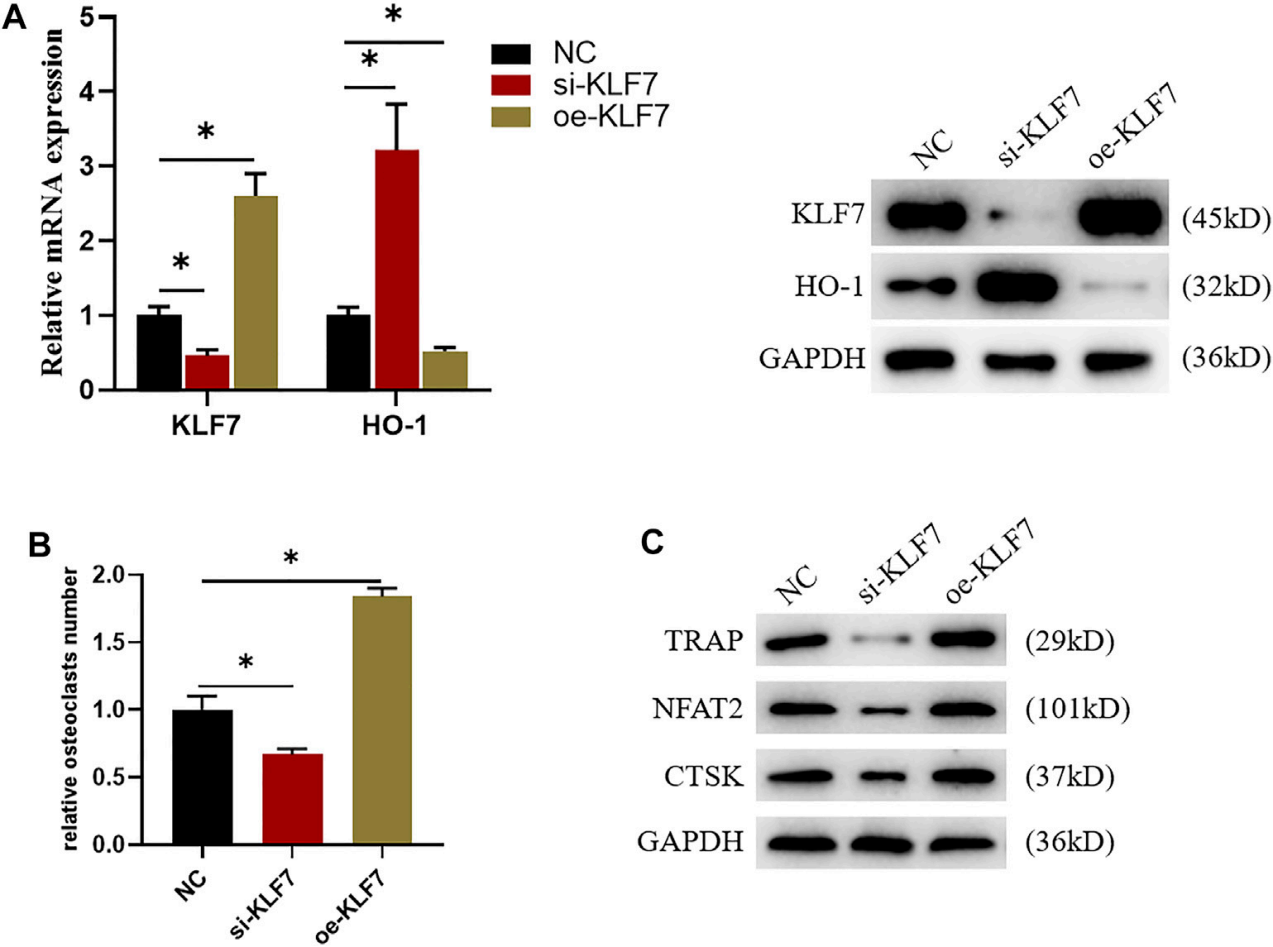
KLF7 can regulate osteoclast differentiation. **(A)** Protein and mRNA expression levels of KLF7 and HO-1 after si-KLF7 and oe-KLF7 treatment. **(B)** TRAP staining showed that macrophage differentiation could be affected by silencing and overexpressing KLF7. **(C)** Changes of the expression levels of osteoclast markers after KLF7 silence and overexpression. *p < 0.05.

Underlying the results of bioinformatics analysis, we speculated that KLF7 could also influence osteoclast differentiation. In support to our speculation, we first constructed si-KLF7 and oe-KLF7 plasmids to processed the cells. The outcome indicated that the knockdown of KLF7 expression led to a marked decrease in mRNA and protein expressions of KLF7, whereas it had an opposite impact on those of HO-1 (Figure 3A). Besides, the high expression of KLF7 dramatically elevated the expression of mRNA and protein expressions of KLF7 while it resulted in a remark decrease in those of HO-1 (Figure 3A). Additionally, the result of TRAP staining exhibited that the knockdown of KLF7 expression attenuated the differentiation ability of osteoclasts, whereas the high expression of this gene strengthened such ability (Figure 3B). Finally, we carried out western blot assay to examine osteoclast-related protein expressions, suggesting that KLF7 affected the expression of osteoclast markers protein (Figure 3C). In sum, the above results suggested that KLF7 could negatively regulate the expression of HO-1, and KLF7 could promote osteoclast differentiation.

### 3.4 KLF7 Constrains Osteoclast Differentiation by Down-Regulating HO-1

**FIGURE 4:**
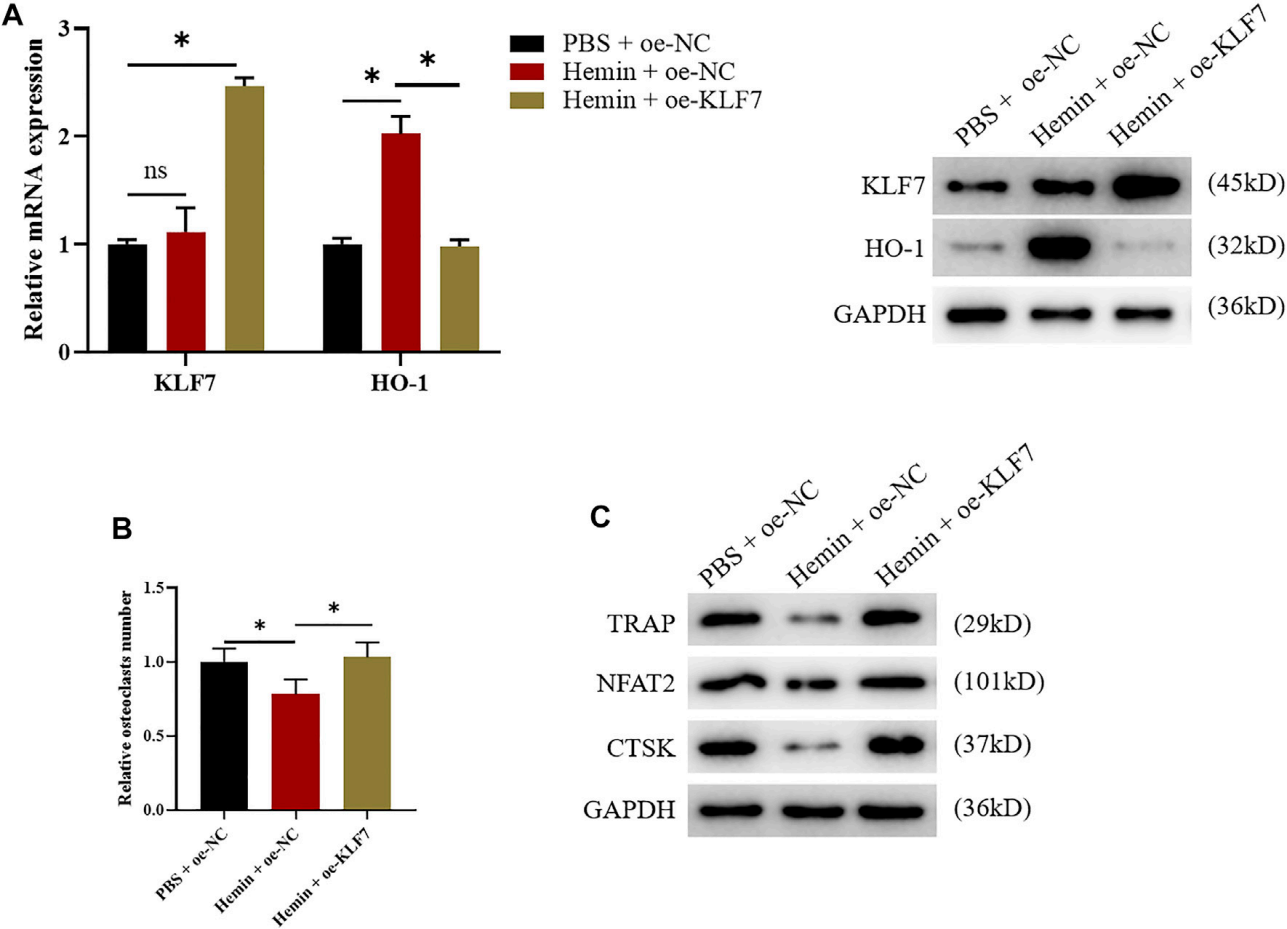
KLF7 promotes osteoclast differentiation by suppressing HO-1 expression. **(A)** qPCR and western blot results showed changes of KLF7 and HO-1 expressions after hemin treatment and KLF7 overexpression. **(B)** TRAP staining showed osteoclast differentiation regulated by HO-1 and KLF7. **(C)** Expression levels of osteoclast markers measured by western blot assay. *p < 0.05.

After the possible effects of KLF7 on osteoclast differentiation were clear, we further validated the binding relationship between TF KLF7 and HO-1. First of all, we separately used hemin and oe-KLF7 to process the osteoclasts. Hemin enhanced HO-1 expression, whereas oe-KLF7 could reverse this effect. (Figure 4A). Based on the above results, we respectively used hemin and oe-KLF7 to process RAW264.7 cells, revealing that hemin notably inhibited osteoclast differentiation. However, after the overexpression of KLF7, this inhibited effect was attenuated (Figures 4B,C). Taken together, the above results indicated the regulatory relationship between KLF7 and HO-1 that KLF7 suppressed HO-1 expression to facilitate osteoclast differentiation.

## 4 Discussion

The clinical treatment of osteoporosis mainly includes calcium and vitamin-based nutrients, estrogen receptor modulators, disphosphonates, and anti-RANKL drugs, etc. (Qaseem et al., 2017). Although the above drugs can enhance bone weight of osteoporosis patients, the prevention and treatment of fractures are not ideal (Sozen et al., 2017). With the breakthrough of targeted drugs in the treatment of cancer, this kind of drugs were gradually applied in the treatment of other diseases. For example, the anti-RANKL desulumab is an emerging anti-osteoporosis targeted drug, which also shows excellent clinical effects (Lewiecki, 2018). Therefore, exploring the molecular mechanism that affects the pathogenesis of osteoporosis and possible effective therapeutic molecular targets are effective ways to develop new treatments and drugs for osteoporosis.

HO-1 is a key gene that is usually found to affect the damage repair of the bones. Many studies have investigated the impact of HO-1 on osteoporosis. Studies proved that osteoclast differentiation is among various factors that affect osteoporosis (Blangy et al., 2020). For example, the occurrence of adolescent Paget’s bone disease is induced by the overactivity of osteoclasts caused by heredity (Masi et al., 2015; Palagano et al., 2018). Besides, in research relating to the influence of HO-1 on osteoporosis, researchers revealed that thorn protein can activate Nrf2/HO-1 signaling pathway to obstruct the formation of osteoclast (Liu et al., 2021). Li et al. (2018) found that lutein inhibits oxidative stress and inflammatory response in osteoporosis model via regulating Nrf2/HO-1, thus suppressing the development of this disease. Herein, it was found that HO-1 could inhibit the osteoclasts differentiation and osteoporosis in mice, which was consistent with the results of previous studies.

The regulation of TF is the commonest regulatory form of gene expression, and the related regulatory relationship of transcription factor is regarded as a key factor that affects the occurrence of osteoporosis. For instance, TFs like AP‐1 and Mitf can impact the activity of NF-kB pathway by modulating NFATc1 and then induce macrophage to differentiate into osteocyte (Pang et al., 2019). Moreover, the osteoclasts differentiation was also considered to be related to the expression of TFs. Recent studies discovered that the TF SREBP2 can promote osteoclast differentiation by affecting the expression of RANKL (Inoue and Imai, 2014). Through bioinformatics analysis, miR-204-5p was found as a key regulator to alter bone health condition in the elderly, while KLF7 was identified as a target gene of miR-204-5p (Chen et al., 2017). Similarly, by utilizing bioinformatics approaches and ChIP assay, this study found that TF KLF7 could bind HO-1 and their expression was negatively correlated. Thereafter, TRAP staining and western blot unveiled that KLF7 promoted the expression of osteoclast markers and osteoclast differentiation. KLF7 is also found to be involved in the progression of diseases. For instance, in research relating to diabetes, KLF7 can directly bind to the promoter region in IL-6 and accelerate the overexpression of IL-6 in inflammatory signaling TLR4/NF-kB/IL-6, thereby causing inflammation (Wang et al., 2017; Zhang et al., 2018). Generally, it is well-accepted that KLF7 exerts its effect on promoting the transcription of downstream genes in the cells. However, in recent years, researchers discovered that the KLF family can also inhibit the expression of target genes by blocking transcription cofactors. For example, Tang et al. (2021) found that KLF12 can bind to the promoter of ENO2 and transcriptionally inhibit the expression of ENO2. Kong et al. (2018) reported that KLF4 can transcriptionally inhibit the expression of STK33 in gastric cancer cells. Therefore, we speculated that KLF7, which belongs to the KLF family, also showed the characteristic of inhibiting the transcription of downstream gene. Herein, similar regulation was unveiled, namely, KLF7 suppressed the expression of the downstream gene HO-1thereby promoting osteoclasts differentiation. This regulatory axis in osteoporosis was first discovered in this study.

The results of this study confirmed that the TF KLF7 could induce osteoclast differentiation by inhibiting HO-1, thereby promoting the development of osteoporosis. Our study provides a novel possible target for the development of osteoporosis drugs, which is of great clinical value. Although cell experiments have proved the role of KLF7 in osteoclast differentiation, but it has not been verified at animal level, which is a shortcoming of this study. Therefore, we plan to carry out further animal experiments to further explore the therapeutic value of KLF7 in osteoporosis.

## Data Availability

The original contributions presented in the study are included in the article/Supplementary Material, further inquiries can be directed to the corresponding author.
